# Deep Sequencing–Based Transcriptome Profiling Reveals Comprehensive Insights into the Responses of *Nicotiana benthamiana* to *Beet necrotic yellow vein virus* Infections Containing or Lacking RNA4

**DOI:** 10.1371/journal.pone.0085284

**Published:** 2014-01-09

**Authors:** Huiyan Fan, Haiwen Sun, Ying Wang, Yongliang Zhang, Xianbing Wang, Dawei Li, Jialin Yu, Chenggui Han

**Affiliations:** State Key Laboratory for Agrobiotechnology and Department of Plant Pathology, China Agricultural University, Beijing, China; The Ohio State University/OARDC, United States of America

## Abstract

**Background:**

*Beet necrotic yellow vein virus* (BNYVV), encodes either four or five plus-sense single stranded RNAs and is the causal agent of sugar beet rhizomania disease, which is widely distributed in most regions of the world. BNYVV can also infect *Nicotiana benthamiana* systemically, and causes severe curling and stunting symptoms in the presence of RNA4 or mild symptoms in the absence of RNA4.

**Results:**

Confocal laser scanning microscopy (CLSM) analyses showed that the RNA4-encoded p31 protein fused to the red fluorescent protein (RFP) accumulated mainly in the nuclei of *N. benthamiana* epidermal cells. This suggested that severe RNA4-induced symptoms might result from p31-dependent modifications of the transcriptome. Therefore, we used next-generation sequencing technologies to analyze the transcriptome profile of *N. benthamiana* in response to infection with different isolates of BNYVV. Comparisons of the transcriptomes of mock, BN3 (RNAs _1+2+3_), and BN34 (RNAs _1+2+3+4_) infected plants identified 3,016 differentially expressed transcripts, which provided a list of candidate genes that potentially are elicited in response to virus infection. Our data indicate that modifications in the expression of genes involved in RNA silencing, ubiquitin-proteasome pathway, cellulose synthesis, and metabolism of the plant hormone gibberellin may contribute to the severe symptoms induced by RNA4 from BNYVV.

**Conclusions:**

These results expand our understanding of the genetic architecture of *N. benthamiana* as well as provide valuable clues to identify genes potentially involved in resistance to BNYVV infection. Our global survey of gene expression changes in infected plants reveals new insights into the complicated molecular mechanisms underlying symptom development, and aids research into new strategies to protect crops against viruses.

## Introduction

Rhizomania is a soil-borne disease caused by *Beet necrotic yellow vein virus* (BNYVV) and is a major threat to sugar beet production throughout the world [1]. BNYVV has a multipartite positive-sense, single-stranded RNA genome and is transmitted by the soil-inhabiting plasmodiophorid *polymyxa beate* [2], [3]. In general, all natural isolates of BNYVV consist of at least four RNA species, although some isolates contain a fifth RNA. RNA1 and RNA2 are sufficient to establish mechanical infections of some experimental hosts, and encode genes necessary for replication, encapsidation, cell-to-cell movement, silencing suppression, and vector transmission [4]–[6]. The smaller RNAs (RNAs 3, 4, and 5) have beneficial roles in natural infections. The P25 protein, which is encoded by RNA3 shuttles between the nucleus and the cytoplasm [7], and is required for development of rhizomania symptoms in sugar beet and a severe local lesion phenotype in some hosts [8], [9]. Compared with RNA3, RNA4 has minor effects on leaf symptoms on *Tetragania expansa* and some *Beta* species [10], but RNA4-encoded p31 is required for efficient vector transmission and root-specific suppression of virus-induced gene silencing [10], [11]. RNA5, which encodes a 26-kDa protein that can influence symptom severity by acting synergistically with RNA3 [12]. BNYVV can infect *Nicotiana benthamiana* systemically and elicits severe or mild symptoms depending on different combinations of viral RNAs [11], [13]. During systemic infection of *N. benthamiana*, RNA4 is highly stable and can induce severe symptoms such as curling and shunting, but RNA3 does not affect these symptom phenotypes [11], [14]. Until now, a major focus in studies of BNYVV pathogenicity has centered on functional analyses of the RNA3-encoded p25 protein [7], [8], [9]. However, little is known about physiological alterations exhibited by the host in response to BNYVV infection, or the molecular mechanisms associated with the severe, RNA4-specific symptoms observed in *N. benthamiana*.

Over the past decade, several differential screening techniques have been used to identify changes in the expression of host genes induced during virus infection. These techniques range from *in situ* hybridization with individual genes to genome-wide transcriptional profiling using oligonucleotide or cDNA microarrays. Recently, next-generation deep-sequencing techniques, such as Solexa/Illumina RNA-seq, have provided new approaches to study the transcriptome. Transcriptome analysis using this short-read high-throughput sequencing technology is more sensitive for detection of low-abundance transcripts than traditional microarray hybridizations [15], [16], and is not restricted to the genomes of model organisms [17], [18]. Transcriptome analyses of some virus-infected plants, such as *Arabidopsis thaliana*, *N. benthamiana*, tomato and rice, have identified gene expression patterns and metabolic networks that are correlated with development of disease symptoms [19]–[24]. Several studies have characterized transcription in transgenic *A. thaliana* plants that constitutively express the BNYVV p25 protein, as well as sugar beet plants infected with virus from the fungal (*P. betae*) vector. These experiments have used either microarray analysis [25] or restriction fragment differential display polymerase chain reaction (RFDD-PCR) [26]. However, none of the studies have investigated the transcriptome response to BNYVV in the model plant *N. benthamiana*. Unlike *A. thaliana*, *N. benthamiana* is able to support replication of BNYVV [11], [13] and this enables comparative analyses to uncouple the effects of infection with BNYVV from those induced by the *P. betae* vector. *N. benthamiana* is one of the most widely-used experimental hosts in plant virology [27]. The recent release of the draft genome sequence for *N. benthamiana* consolidates its role as a model to investigate plant-pathogen interactions and to compare gene expression between different plant-pathogen pairs [22], [27]. Methods for transient overexpression or gene silencing, as well as facile expression of fluorescent protein fusions by agroinfiltration or viral vectors for protein–localization studies have made *N. benthamiana* an increasingly attractive host to study functional genomics [27], [28]. Furthermore, *N. benthamiana* has been used successfully to study interactions between various immune receptors and pathogen effectors as well as immune signaling [29]. Owing to the technical limitations of microarrays, very few studies at the global transcriptome level have been reported in response to viral infection in *N. benthamiana*. However, recently developed next-generation deep-sequencing techniques, which have clear advantages over microarray analysis, have provided new approaches to study the transcriptome [29], [30].

We have used deep RNA sequencing (Solexa/Illumina RNA-Seq) to investigate the genetic architecture of the *N. benthamiana* transcriptome and to analyze the gene expression changes induced by BN3 (RNAs _1+2+3_) and BN34 (RNAs _1+2+3+4_). Our results provide insights into the mechanisms responsible for appearance of disease symptoms and to best of our knowledge, this is the first report to study *N. benthamiana* responses to virus infection at the whole transcriptome level.

## Results and Discussion

### Subcellular localization of the p31 protein from BNYVV RNA4 in *N. benthamiana*

**Figure 1:**
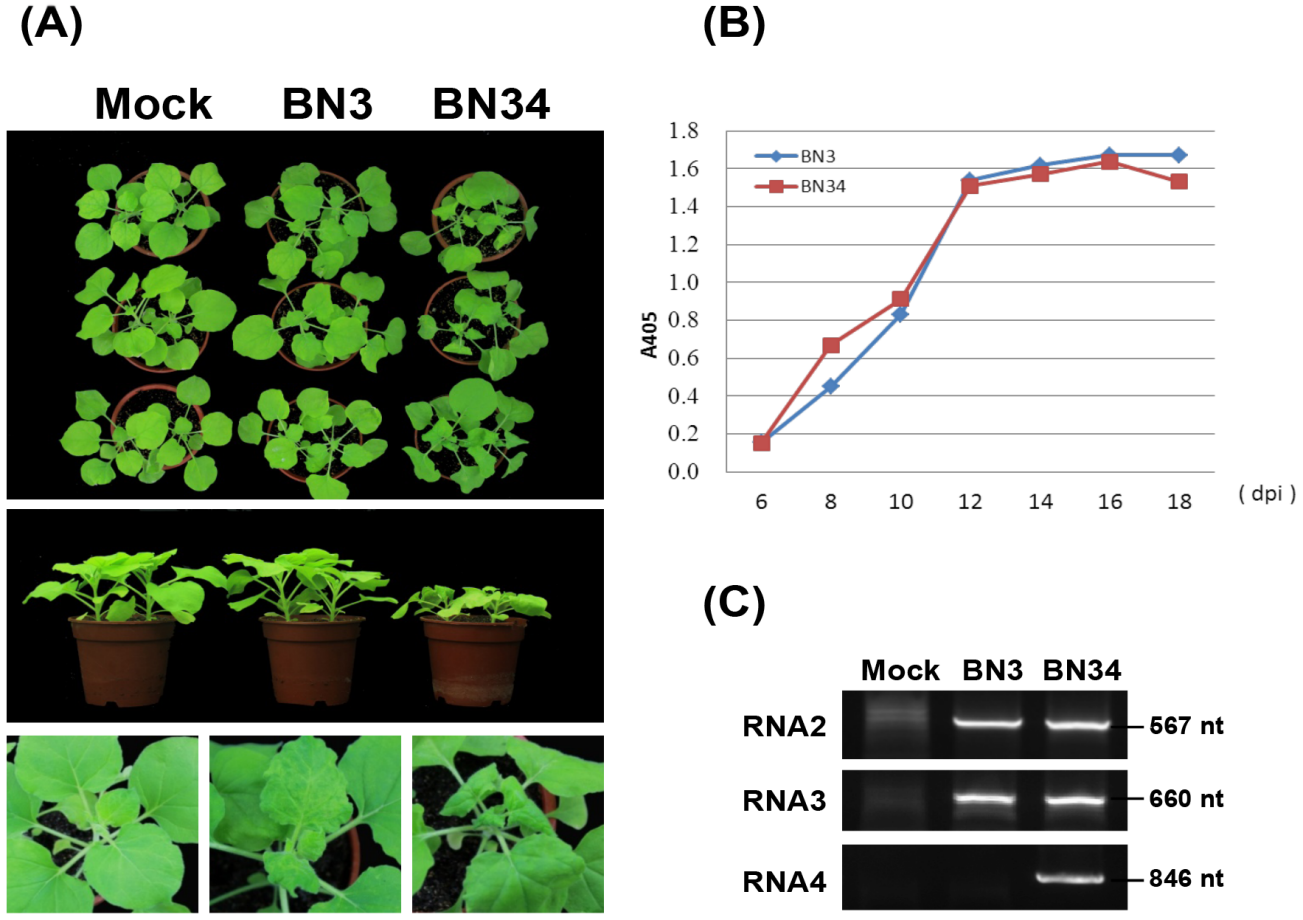
BNYVV phenotypes and detection of virus in systemically infected *N. benthamiana* leaves. (**A**) Systemic symptoms elicited in *N. benthaliana* plants by BN3 and BN34. Mock-infection shows plants rubbed with buffer. (**B**) Time course of BNYVV accumulation in *N. benthamiana* systemically infected leaves at 6, 8, 10, 12, 14, 16, 18 days post-inoculation. The two curves in the graph represent ELISA analyses of BN3 and BN34 samples. (**C**) RT-PCR amplification of RNA2, RNA3, and RNA4 from systemically infected leaves of *N. benthamiana*. The sizes of the PCR fragments are indicated on the right.

Preliminary experiments have indicated that BNYVV can infect *N. benthamiana* systemically and the different RNA combinations elicit severe or mild symptoms [12]. Additional experiments revealed that the severe symptoms were associated with the presence of the root-specific suppressor p31 encoded by RNA4 [11]. Consistent with previous studies, our results have shown that the Chinese isolate BN34 induces very severe symptoms, including stunting and downward curling of the upper leaves by 12–14 days post-inoculation (dpi), whereas infection by BN3 results in mild symptoms in *N. benthamiana* (Figure 1A).

**Figure 2:**
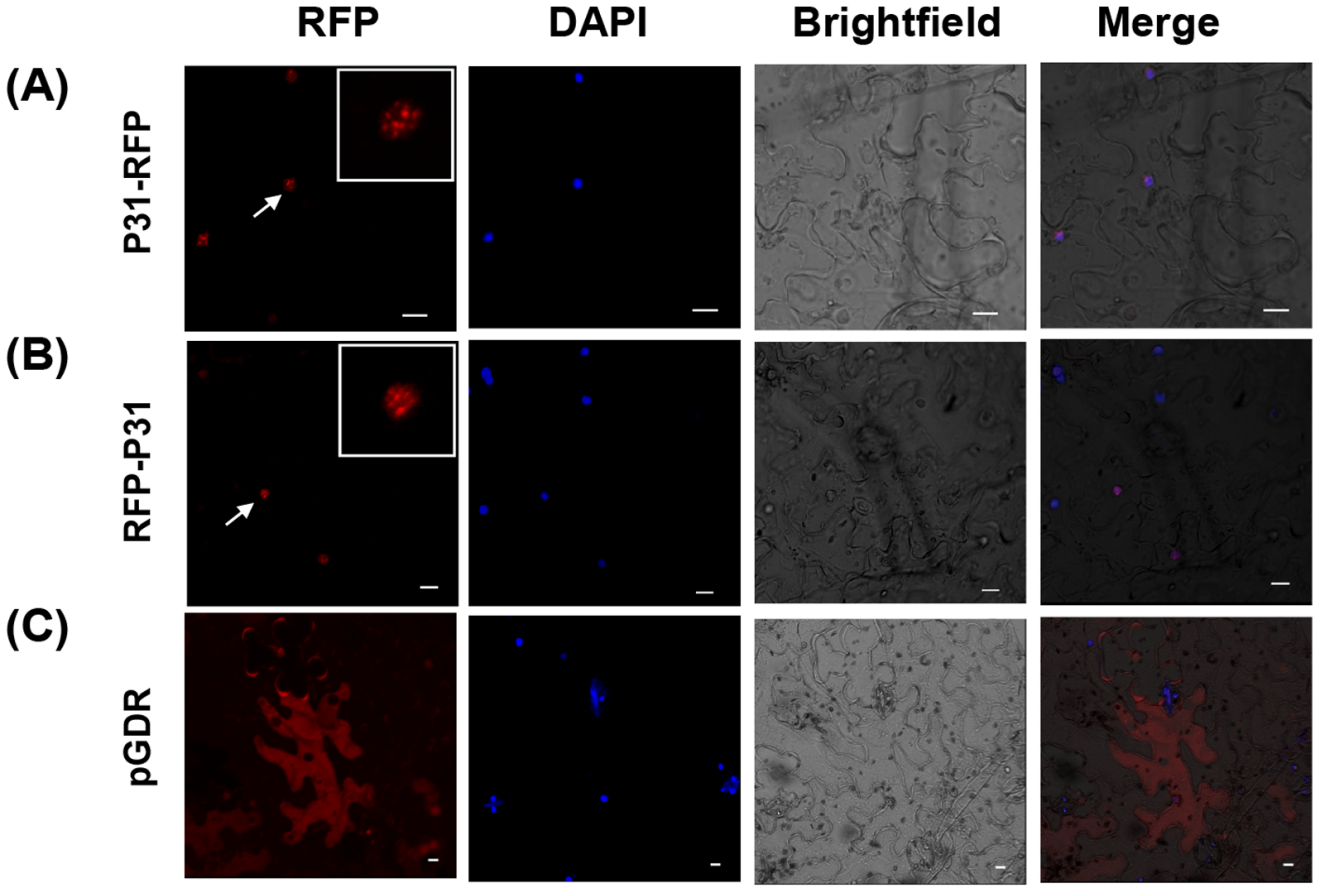
Subcellular localization of BNYVV p31 expressed in *N. benthamiana* leaves, as determined by fluorescence microscopy of RFP-expressing plant tissue. Epidermal leaf cells agro-infiltrated with (A) P31-RFP and (B) RFP-P31 vectors for transient expression of fusion proteins. (C) The pGDR binary vector expressing only RFP was infiltrated in parallel as a control. RFP fluorescence was used to evaluate expression, and DAPI (4, 6-diamidino-2-phenylindole) was used for nuclear localization in leaf cells. Co-localization of the nuclei and p31 is shown in the merged panel. Arrows in the images identify locations of the magnified inset panel highlighting the nuclei. Bars = 10 µm.

Several viral proteins with the capacity to suppress RNA silencing are targeted to the nucleus; these include the p19 suppressor protein of *Tomato bushy stunt virus*, the 2b protein of *Cucumber mosaic virus*, and the P0 protein of *Beet western yellows virus* [31]–[33]. In order to determine the subcellular localization of the p31 protein, plasmids harboring the p31protein fused with the red fluorescent protein (RFP) at the C-terminus (P31-RFP) or the N-terminus (RFP-P31) were transiently introduced into *N. benthamiana* leaf cells via agro-infiltration. Confocal fluorescence microscopy analyses at 3 dpi revealed punctate red fluorescent foci restricted mainly in the nucleus, and these foci co-localized with the 4′-6-diamidino-2-phenylindole dihydrochloride (DAPI) signal, but no fluorescence was apparent in the cytoplasm (Figure 2). As a negative control, RFP resulted in a diffuse pattern of fluorescence in both the cytoplasm and nuclei, and this indicated that both the P31-RFP and RFP-P31 fusions localize inside the nuclei of *N. benthamiana* leaf cells. The nucleus is a complex, highly structured organelle that is responsible for gene activation, repression and expression [34], [35], so this finding led us to speculate that nuclear localization of p31 might be linked to elicitation of disease symptoms by interacting with nuclear elements that affect the host transcription network and to network changes leading to a series of physiological changes.

### Illumina sequencing and assembly of sequencing reads

To determine the best time to analyze transcriptional responses to systemic BNYVV infection, an ELISA time-course analysis of viral accumulation was performed. The levels of BNYVV coat protein increased for the first 12 days and then leveled off, but remained relatively constant for the next 6 days (Figure 1B). Similar to a previous study [11], our ELISA analysis shows that there are no dramatic differences in virus accumulation in BN3 and BN34-infected plants. This indicates that accumulation of BNYVV does not correlate with symptom severity in *N. benthamiana*. Young aerial expanding leaves and apices (shoots) were collected at 12 dpi for RNA extractions from BNYVV-infected and mock-inoculated plants. To reduce biological errors caused by natural variation, six inoculated plants that exhibited similar symptoms were harvested to prepare one pooled RNA sample, as has been a protocol in comparable studies [36]–[38]. RT-PCR analysis of the total RNA sample was carried out to confirm the presence of BNYVV in systemically infected leaves (Figure 1C), and three cDNA libraries were constructed and used for Illumina deep sequencing. After removing adaptors and reads of unknown or low-quality nucleotides, we obtained 25,185,932, 45,742,778 and 26,731,472 clean reads for mock-, BN3-, and BN34-infected plants, which gave a total of 2,603,430,944 bp, 4,734,216,632 bp, and 2,770,205,174 bp for mock-, BN3-, and BN34-infected plants, respectively. The clean reads were aligned to the *N. benthamiana* draft genome using TopHat (http://tophat.cbcb.umd.edu/) software, resulting in 20,712,030, 36,494,174 and 20,467,702 reads that match either to unique or to multiple genome locations (Table 1). The alignment results were analyzed by Cuffinks software and assembled into transcripts (http://cufflinks.cbcb.umd.edu/). Combination of the sequence data from the three libraries revealed 30,055 transcript assembly contigs (TACs) larger than 100 bp (N50 of 966 bp), with an average GC content of 43.03%. This is very similar to a previous report for the transcriptome of *Verticillium dahliae*-infected *N. benthamiana* [39]. The distribution of the total TAC sizes is shown in Table S1 and further assembly analysis showed that the total TAC assembly consisted of 27,890 unigenes.

**Table 1:** Summary statistics for sequencing and sequence assembly for three libraries prepared from mock-, NB3- and BN34-infected *N. benthamiana* plants.

	Mock	BN3	BN34
**Total base pairs (bp)**	2,603,430,944	4,734,216,632	2,770,205,174
**Total reads**	25,185,932	45,742,778	26,731,472
**Mean length of reads**	99.68	99.40	99.41
**Matched to ** ***N. benthamiana***	20,712,030	36,494,174	20,467,702
	(82.24%)	(79.78%)	(76.57%)
**Unmatched to ** ***N. benthamiana***	4,473,902	9,248,604	6,263,770
	(17.76%)	(20.22%)	(23.43%)
**Matched to BNYVV**	728	1,667,555	1,789,384
	(0.00%)	(3.65%)	(6.69%)

### Functional annotation by sequence comparison with public databases

**Figure 3:**
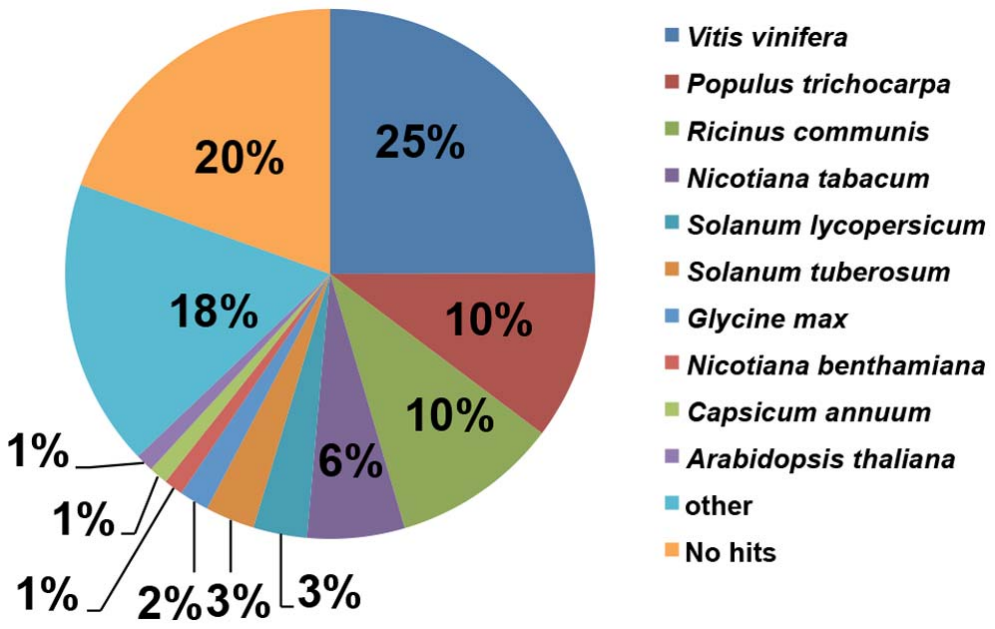
Species distribution of unigene BLASTX results. The figure shows the species distribution of unigene BLASTX results against the NCBI-NR protein database with a cutoff E value ≤10^−5^ and the proportions of each unique species. Different colors represent different species. Species with proportions of more than 1% are shown.

Unigenes functions were annotated by using BLASTX (E-value≤1.0e^−5^) against the public databases (NCBI NR, SwissPort, GO, COG and KEGG) (File S1; See supporting information). A total of 24,024 unigenes were matched to one or more of the databases (Table S2; See supporting information). This indicates that Illumina paired-end sequencing detected expression of a large proportion of the diverse genes expressed in *N. benthamiana*. However, some unigenes were obtained only from BNYVV-infected or non-infected samples. We believe that these differences may be caused by long-term ecological adaptation to virus infection. The species distributions of the best-match result for each sequence are shown in Figure 3. It was surprising that *N. benthamiana* shared the highest similarity (25%) to the best-match homologs with the grape (*Vitis vinifera*) in the BLASTX annotation, followed by 10% for each of *Populus trichocarpa* and *Ricinus communis*.

GO analyses were used to classify functions of predicted *N. benthamiana* unigenes. The unigenes were assigned to three main categories (biological processes, cellular components, and molecular functions), which together contain 34 subcategories (Figure S1; See supporting information) that provide a good indication of the diversity of genes affected by viral infection. Similarly, COG annotation yielded approximately 7,967 putative proteins in 25 clusters (Figure S2; See supporting information). Among these categories, the cluster for “general function prediction only” was the largest group, followed by “translation, ribosomal structure and biogenesis” and “posttranslational modification, protein turnover, chaperones”.

The annotations by KEGG also provide insight into the various biological pathways associated with viral infection. A total of 9,272 unigenes were distributed in 162 KEGG pathways. The top three KEGG pathways containing the largest numbers of unigenes include “metabolic pathways”, “biosynthesis of secondary metabolites” and “microbial metabolism in diverse environments” (Table S3; See supporting information). The unigenes information will be very useful for future genome annotation of *N. benthamiana* and will contribute to the identification of novel genes involved in the response pathways to plant virus infection.

### Global changes of transcript expression in response to BNYVV infection

**Figure 4:**
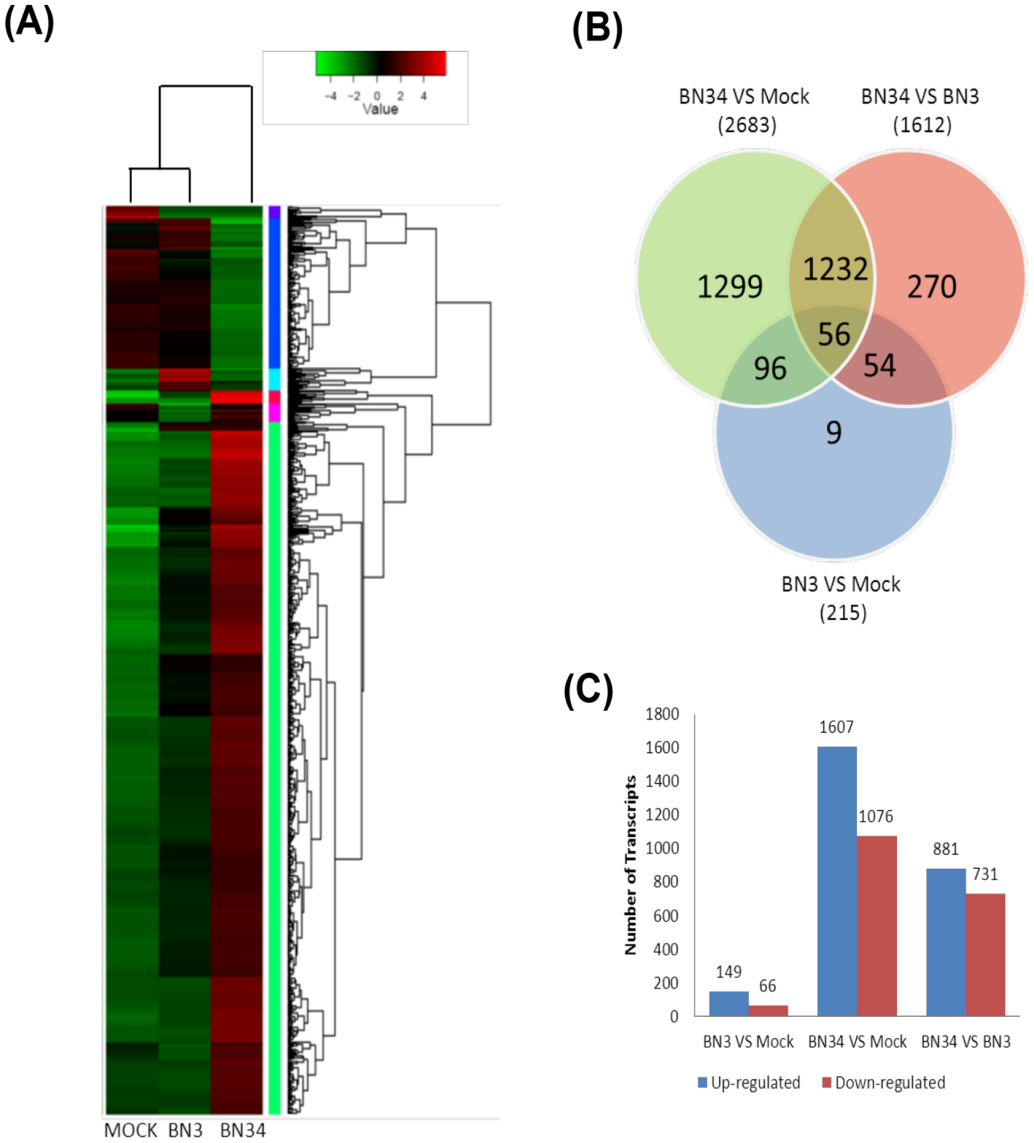
Summary of differentially expressed genes among the three libraries. (A) Hierarchical clustering of differential expression profiles for 2,976 genes among mock-, BN3-, and BN34-infected plant libraries was based on log ratio FPKM data (FDR≤0.05 and absolute value of the log_2_ ratio ≥2). Green represents lower expression, red represents high expression, the column represent individual experiments and the rows represent transcriptional units. (B) Venn diagram showing differentially expressed genes that are unique or shared among three libraries of mock-, BN3-, and BN34-infected plants. BN3 VS mock refers to comparisons between BN3-infected and mock-inoculated libraries. BN34 VS mock shows comparisons between BN34-infected and mock-inoculated libraries. BN34 VS BN3 refers to comparisons between BN34-infected and BN3-infected libraries. The numbers of differently expressed genes are shown in each section of the figure. (C) Changes in gene expression profiles among the mock-, BN3-, and BN34-infected plant libraries. The numbers of up-regulated and down-regulated genes between the mock and BN3, mock and BN34, BN3 and BN34 are summarized.

To identify differentially expressed sequences accumulating in response to BNYVV infection, we measured gene expression levels in fragments per kilobase of exon per million fragments mapped (FPKM) and used a false discovery rate (FDR) ≤0.05 as a threshold to judge the statistical significance of transcript expression. According to the FPKM method, 3,016 differentially expressed transcripts were detected in two-way comparisons of the three libraries (File S2; See supporting information). Comparisons of mock- and BN3-infected plants (BN3 VS mock) revealed 215 significantly altered transcripts, with 149 up-regulated and 66 down-regulated transcripts. The mock- and BN34-infected comparisons (BN34 VS mock) generated 2,683 altered transcripts, with 1,607 up-regulated and 1,076 down-regulated by infection. BN3- versus BN34-infected plants (BN34 VS BN3) revealed expression changes in 1,612 transcripts, including 881 up-regulated and 731 down-regulated genes. In addition, there were 113, 85, and 105 transcripts that were uniquely expressed in the libraries of mock-, BN3- and BN34-infected plants. Changes in the abundances of transcripts among the three groups of mock-, BN3-, and BN34-infected plants are represented in a Venn diagram and by hierarchical clustering (Figure 4 A and B). The number of genes regulated in response to BN34 infection is approximately 12-fold greater than these of BN3 infection, with only 0.5% of co-regulated genes. This indicates that there is a greater transcript difference between severe and mild infection than between infected and non-infected plants. Moreover, the number of genes altered by each virus isolate was consistent with the severity of the symptoms observed. Similar results were reported previously using less comprehensive methods for responses of *N. benthamiana* to other viruses [20], [23].

### Functional analysis of differentially expressed transcripts

**Figure 5:**
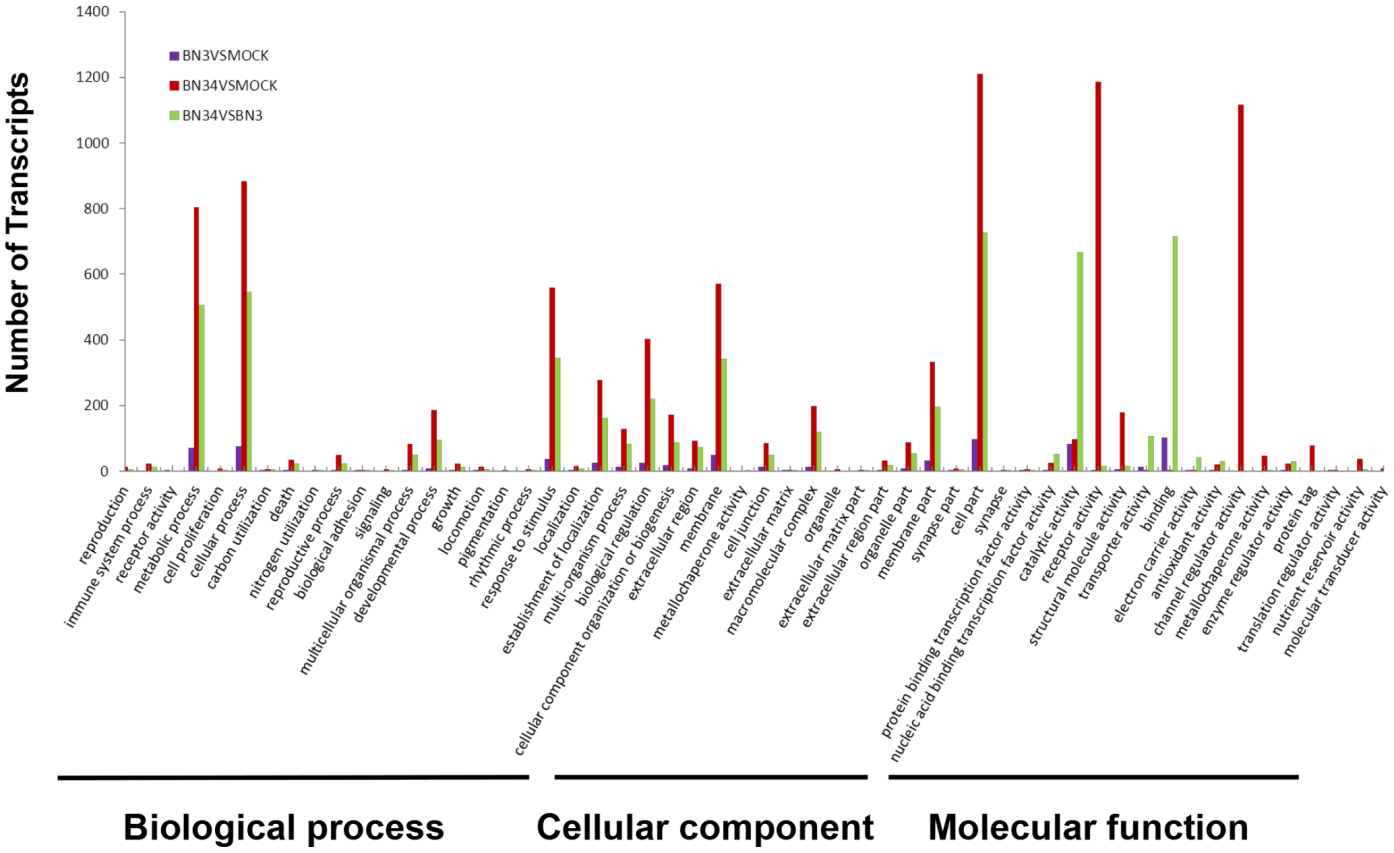
Analysis of gene ontology (GO) functional enrichment of differentially expressed BN3 VS mock, BN34 VS mock, and BN34 VS BN3 genes. The functions of genes identified cover three main categories: biological processes, cellular components, and molecular functions.

GO enrichment analyses were performed to classify the putative functions of differentially expressed transcripts in two-way comparisons of libraries prepared from mock-, BN3-, and BN34-infected plants. Based on sequence homologies, the differentially expressed transcripts were separated into three main categories (cellular components, molecular functions and biological processes), which included 14, 16, and 24 functional groups, respectively. Among these groups, the BN3 VS mock and BN34 VS mock comparisons had similar distributions of gene functions in the biological processes and cellular component categories. However, within the molecular function category, “receptor activity” and “channel-regulated activity” were significantly enriched in the BN34 VS mock comparison, whereas “catalytic activity” and “binding” were primarily enriched in the BN34 VS BN3 comparison. We also noted that a high percentage of transcripts in both the BN34 VS mock and BN34 VS BN3 comparisons fell into the following functional groups: “metabolic processes”, “cellular processes”, “response to stimuli”, “membranes” and “cell parts” (Figure 5). The GO functional enrichment analysis indicates that the changes in the molecular function may be very important in BNYVV infections.

To further understand the functions of differentially expressed transcripts in response to BN3 and BN34 infections, the differentially expressed sequences were mapped to the KEGG database categories and compared with the whole transcriptome background. We found that a total of 11 pathways were affected by infection with BN3 or BN34, and that of these, six pathways with P-values ≤0.05 were significantly enriched (Table 2). Four common KEGG pathways were significantly enriched after infection with either BN3 or BN34. These are “biosynthesis of secondary metabolites”, “amino sugar and nucleotide sugar metabolism”, “plant-pathogen interaction”, and “galactose metabolism”. However, the “metabolic pathways” and “photosynthesis-antenna proteins” were influenced significantly only by BN34 infection, indicating that genes falling into these two KEGG pathways may contribute to the severe symptoms elicited by viral RNA4 in *N. benthamiana*.

**Table 2:** Significantly enriched KEGG pathways induced in *N. benthamiana* in response to BNYVV infection.

	Enriched P-value^a^		
Pathway category	BN3 VS Mock	BN34 VS Mock	BN34 VS BN3
**Metabolic pathways**	9.50E-01	0.00	0.00
**Biosynthesis of secondary metabolites**	2.91E-03	2.17E-05	1.74E-09
**Plant-pathogen interaction**	5.60E-03	1.09E-06	1.54E-03
**Amino sugar and nucleotide sugar metabolism**	9.57E-04	3.56E-03	3.57E-03
**Photosynthesis - antenna proteins**	-	1.81E-04	4.32E-10
**Galactose metabolism**	7.46E-04	9.11E-03	-

^a^ Only pathways with a p value of ≤0.05 were considered as significantly enriched in the table.

### Confirmation of Solexa expression patterns by qRT-PCR

**Figure 6:**
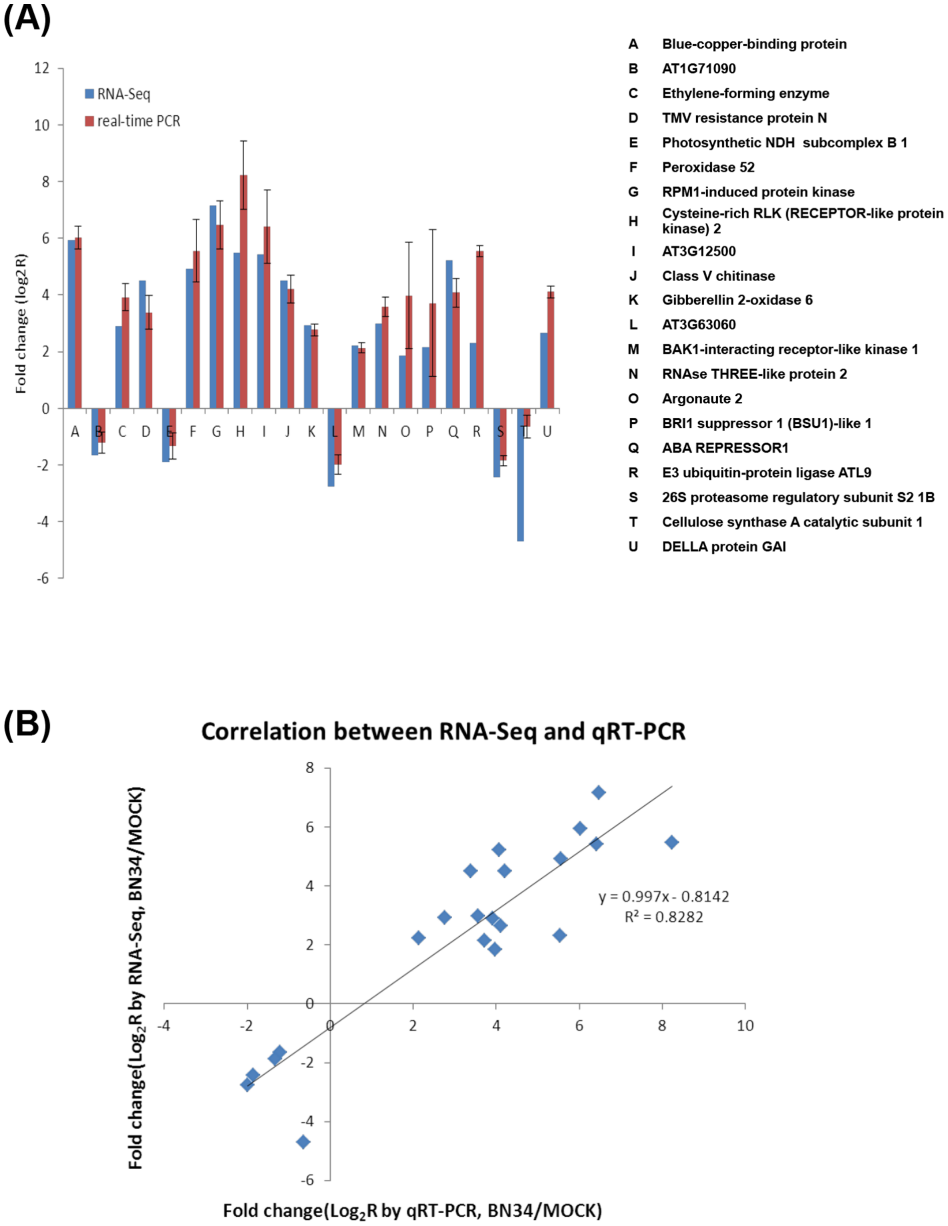
Verification of the relative expression levels of genes by quantitative RT-PCR (qRT-PCR). (A) Expression patterns of selected *N. benthamiana* genes in response to BN34, as determined by qRT-PCR (Red) and RNA-Seq (Blue). The x-axis shows the annotations of selected genes. The y-axis shows the normalized expression levels of the transcripts. Normalization of expression was performed using the *PP2A* gene as internal reference. (B) Correlation of the expression ratio of selected genes measured by qRT-PCR and RNA-Seq.

RNA-Seq revealed the expression profiles of thousands of genes. In order to test the reliability of Solexa sequencing, 26 unigenes with annotations were selected randomly for qRT-PCR analysis using specific primers (Table S4; See supporting information). The results show that 21 of 26 unigenes (80.77%) examined have the same expression profiles as deduced from the original Solexa sequencing (Figure 6A). Inconsistencies among the remaining five genes could be artificially caused by mutations within primer sites or possibly a lower sensitivity of qRT-PCR than RNA-Seq [17], [30]. In addition, a high correlation (R^2^ = 0.8282) was found between RNA-seq and qRT-PCR (Figure 6B). These results suggest that the alterations in gene expression detected by RNA-Seq reflected the actual transcriptome differences between mock-inoculated and BNYVV-infected plants.

### Host responses to BNYVV infection in RNA silencing and ubiquitin-proteasome pathways

RNA silencing and ubiquitin-proteasome pathways are two important systems that plants use for defences against virus infection [40], [41]. RNA silencing is a sequence-specific RNA degradation mechanism, and disease symptoms can be affected by viral silencing suppressors that interfere with cellular processes regulated by RNA silencing[42]. Changes in the expression of genes that encode members of the Argonaute and RNA-dependent RNA polymerase families in response to infection have been observed following infection by many viruses [19], [43], [44]. In our transcriptome data, altered expression of *AGO2* was observed in both BN3- and BN34-infected plants (Table 3 and Figure 6). However, other genes involved in the RNA silencing pathway (*AGO4*, *AGO5*, *AGO10*, *RNase III-like protein 2*) were only affected by BN34 infection. Among these genes, *AGO5* and *RNAse III -like protein 2* were up-regulated, whereas expression of *AGO4* and *AGO10* was reduced. We plan to investigate relationship between these genes expression influences and the resistance of plants to BNYVV in further investigations.

**Table 3:** Expression patterns of genes involved in RNA silencing and ubiquitin-proteasome pathways.

		BN3-infection		BN34-infection	
Gene ID	Description	FC^a^	q-value^b^	FC	q-value
**RNA silencing**					
XLOC_001896	Argonaute 5	0.96	1.92E-01	2.57	5.45E-04
XLOC_006832	Argonaute 4	−0.75	2.69E-01	-2.33	1.56E-03
XLOC_011365	Argonaute 10	−0.90	1.78E-01	-2.08	2.81E-03
XLOC_025131	Argonaute 2	1.67	1.34E-02	1.84	6.70E-03
XLOC_023632	RNAse three-like protein 2	0.26	7.65E-01	2.98	5.45E-05
**Ubiquitin-proteasome**					
XLOC_003292	26S proteasome regulatory subunit S2 1B	0.38	5.78E-01	−2.42	1.44E-03
XLOC_018219	26S proteasome regulatory subunit 4-A	−0.20	8.41E-01	−5.80	1.41E-03
XLOC_003245	E3 ubiquitin-protein ligase ATL9	0.68	3.28E-01	2.31	1.19E-03
XLOC_004288	Ubiquitin-specific protease 14	0.47	5.69E-01	2.44	1.43E-03
XLOC_019444	Ubiquitin-conjugating enzyme E2 A	−6.80	5.79E-05	−6.41	2.18E-04
XLOC_020943	E3 ubiquitin-protein ligase ORTHRUS 2	−3.12	2.81E-03	−3.93	3.04E-03
XLOC_024409	Ubiquitin C-terminal hydrolase 3	0.77	3.71E-01	2.33	4.45E-03
XLOC_024830	E3 ubiquitin-protein ligase SINAT2	4.73	5.65E-02	7.73	3.45E-07
XLOC_024999	Ubiquitin-conjugating enzyme E2 28	0.09	9.03E-01	3.32	4.23E-06
XLOC_026836	polyubiquitin 14	0.17	7.95E-01	2.04	2.73E-03
XLOC_027267	Ubiquitin-associated (UBA) zinc-finger	1.21	9.24E-02	2.54	4.75E-04
XLOC_022324	Senescence-associated E3 ubiquitin ligase 1	2.30	5.99E-03	4.00	1.66E-06

^a^ FC, fold changes(log2 ratio) in gene expression.

^b^ The q-value was false discovery rate.

Recent studies have revealed that the ubiquitin-mediated proteasome pathway is a major regulatory process in several aspects of cell biology, including cell development and pathogen defense [45], [46]. Polerovirus P0 proteins are RNA silencing suppressors that have an F-box motif that allows viruses to usurp host E3 ligases to promote degradation of specific cellular proteins [47], [48]. The P25 protein, encoded by BNYVV RNA3 can interact with a number of candidate proteins involved in ubiquitination to alter target recognition and cause cell necrosis in an as yet undefined manner [49], [50]. Consistent with results from previous expression profiling studies [41], [51], [52], transcription of some genes involved in ubiquitin-proteasome pathways were perturbed significantly in the current study (Table 3). The expressions of genes that encode proteins associated with the 26S proteasome, such as the two 26S proteasome regulatory subunits S2 1B, and 4-A, were suppressed markedly by BN34 infection. However, the trend in expression of genes associated with ubiquitination was not in the same direction. Expression of 7 out of the 10 genes was altered significantly only by BN34 infection. Although it is not known how these genes function in the resistance response, we believe that they may have important roles in virus infection.

### Candidate genes involved in symptom development

Stunting, which is the major symptom observed in *N. benthamiana* plants infected by BN34 [11], commonly results from abnormalities in cell size and shape [53]. Suppression of genes that control the synthesis and structural features of cell walls have been reported for stunted plants infected with certain viruses [22], [53]–[55]. Several pathways determine plant height, but cell-wall-related genes, such as cellulose synthase (*CESA*) genes also play pivotal roles [54], [56], [57]. The cellulose-deficient *Arabidopsis* mutant *rsw3* and CESA-like (CLS)-deficient rice mutant *nd1* display obvious stunting phenotypes [58], [59]. Our RNA-Seq results indicate that genes encoding structural proteins and proteins involved in cell wall assembly are affected in both BN3- and BN34-infected plants. Among these changes, most of the *CESA*-related genes, including the cellulose synthase A catalytic subunit 1 and cellulose synthase A catalytic subunit 2 were suppressed dramatically only after BN34 infection. However, genes associated with other aspects of cell wall composition, such as wall-associated kinase 2, endoglucanase 17, endoglucanase 11, pectinesterase-like protein, pectinesterase, and pectinesterase 7 were altered in different ways following infection with BN3 and BN34 (Table 4). Hence, the down-regulation of *CESA* genes likely contributed more to stunting symptoms in BN34-infected *N. benthamiana* than altered expression of other genes commonly associated with cell walls compositions.

**Table 4:** Expression patterns of genes related to either cell-wall synthesis or gibberellin metabolism.

		BN3-infection		BN34-infection	
Gene ID	Description	FC^a^	q-value^b^	FC	q-value
**Cell-wall associated**					
XLOC_000085	Cellulose synthase A catalytic subunit 2	−0.89	1.91E-01	−2.32	1.35E-03
XLOC_008050	Cellulose synthase A catalytic subunit 1	−0.32	7.04E-01	−4.71	1.03E-04
XLOC_015926	Expansin A1	0.28	6.73E-01	−2.51	4.06E-04
XLOC_001406	Wall-associated kinase 2	5.17	1.41E-02	8.40	7.83E-09
XLOC_019241	Beta-1,3-glucanase-like protein	−0.76	2.51E-01	−2.37	6.96E-04
XLOC_001547	Endoglucanase 17	−1.23	1.03E-01	−3.71	9.91E-05
XLOC_009230	Endoglucanase 6	0.07	9.19E-01	−2.16	1.69E-03
XLOC_021145	Endoglucanase 11	−1.32	1.12E-01	−6.44	2.18E-04
XLOC_001748	Pectin methylesterase 3	0.82	2.16E-01	2.08	2.23E-03
XLOC_000908	Pectinesterase-like protein	−6.42	2.91E-04	−6.04	1.01E-03
XLOC_015688	Pectinesterase 10	1.45	4.24E-02	2.75	1.87E-04
XLOC_011323	Pectinesterase 7	5.83	2.00E-03	10.80	1.60E-15
**Gibberellins metabolism associated**					
XLOC_024448	Gibberellin 2-oxidase 6	−0.37	6.41E-01	2.70	2.57E-04
XLOC_017491	Gibberellin-regulated family protein 5	1.26	1.31E-01	2.99	1.73E-04
XLOC_024875	DELLA protein GAI	1.08	1.56E-01	2.65	4.93E-04
XLOC_002763	Putative gibberellin receptor GID1L2	0.24	1.00E+00	3.04	3.13E-04

^a^ and ^b^, see Table 4.

Plant height also is influenced by the concentration of gibberellic acid (GA), which promotes cell elongation [60]–[63]. Infection of rice plants with *Rice dwarf virus* (RDV) causes a significant reduction in GA1 levels, and treatment of RDV-infected plants with GA3 is able to restore the non-dwarf phenotype [64]. Interactions of RDV P2 with ent-kaurene oxidase, a key component involved in GA synthesis in plants, has been linked to dwarfing in infected rice [64]. Our study did not reveal a direct influence of GA biosynthesis through demonstrated changes in hormone biosynthesis genes. Nonetheless, we did note a significant increase in the level of transcripts for the gibberellin 2-oxidase protein (Table 4 and Figure 6), which is an enzyme involved in GA catabolism that regulates plant growth through reduction of the endogenous levels of bioactive GAs [65]. Recent research has shown that the rice H032 mutant, which over-expresses *OsGA2ox1* has a dwarf phenotype that resembles symptoms in rice plants infected by either *Rice black streaked dwarf virus* or RDV[66]. In addition, the levels of transcripts encoding the DELLA protein GAI and gibberellin-regulated family protein 5 were increased after BN3 and BN34 infections. These genes are considered to act as negative regulators of the GA signaling pathway in *Arabidopsis* [67]–[69].

It is difficult to determinate which gene(s) make(s) the most important contributions to development of disease symptoms in BN34-infected *N. benthamiana* on the basis of gene expression data alone. Hence, the roles of these key candidate genes in development of symptoms associated with BNYVV infection will be investigated more extensively in the future by using transgenic plants with elevated or reduced expression levels of the target genes.

## Conclusions

The large set of TACs identified in this study represents the first global analysis of the *N. benthamiana* transcriptome in response to BNYVV. The data sets have permitted refined comparisons of mRNA changes in BNYVV-infected and mock-inoculated samples and have identified a suite of candidate genes that respond to virus infection. Our results indicate that suppression of *CESA* genes and decreases in GA accumulation may act in concert to contribute to the stunted growth occurring during infectious containing BNYVV RNA4. In addition, we found that the p31 protein encoded by RNA4 localizes to the nucleus and this subcellular localization may provide a link between changes in the transcriptome and the severity of symptoms. The recently released draft genome sequence of *N. benthamiana* citations will permit us to carry out more specific characterization of the candidate genes. We anticipate that such studies will lead to a better understanding of the multidimensional networks culminating in plant virus symptoms in this model host.

## Materials and Methods

### Plant growth, viral inoculation and detection

*N. benthamiana* plants were grown in a controlled-environment chamber at 24±1°C under a 16-h light and 8-h dark regimen. BN3 (RNAs _1+2+3_) was derived from the BNYVV Hu3 isolate described previously [70], and BN34 (RNAs _1+2+3+4_) was a mixture of total RNAs from the BN3 inoculated leaves of *T. expansa* and *in vitro* transcripts of RNA4. Virus inoculum supplemented with an equal volume of inoculation buffer (50 mM glycine, 30 mM K2HPO4, 1% bentonite, 1% celite, pH 9.2) was rubbed onto *N. benthamiana* leaves. An ELISA time course was performed as described previously [13] with seven time points after inoculation to detect virus content. Total RNA was extracted from mock and virus-infected systemic leaves of *N. benthamiana* for RT-PCR detection at 12 dpi, using TRIzol reagent as described by the manufacturer (Invitrogen, Carlsbad, CA, USA). The concentration and quality of total RNAs were determined on a 1% agarose gel and a spectrophotometer (Nanodrop ND-2000, ThermoFisher Scientific, Wilmington, DE, USA). The primers used for quantification of RNA2, RNA3, and RNA4 by RT-PCR were described previously [14].

### Construction of recombinant plasmids

To obtain a transiently expressed RFP-P31 fusion protein, the full-length p31 gene was amplified with the RFP-P31F and RFP-P31R primers (Table S3) from pUOF1-6, an infectious cDNA clone from BNYVV RNA4 [11]. PCR fragments were digested with *Hin*dIII and *Bam*H I and integrated into the *Hin*dIII- *Bam*HI sites of a pGDR [71] expression vector (kindly provided by Professor Andrew Jackson, University of California at Berkeley) to generate the RFP-P31 recombinant plasmid for expression of the RFP-P31 fusion protein. For the construction of a P31-RFP fusion protein vectors, the DsRed2 coding region with a linker sequence (containing *Hin*dIII, *Sal* I and *Apa* I) was amplified with the RFPF and RFPR primers (Table S3). The resulting fragment was digested with *Hin*dIII and *BamH* I, and cloned into vector pGD [71] to generate the plasmid pGD-RFP. The primers P31-RFPF and P31-RFPR (Table S3) were used to amplify the p31 sequence, which was further cloned into pGD-RFP using *Hin*dIII and *Sal* I restriction enzymes to generate the construct P31-PFP. The sequences of all recombinant plasmids were verified by DNA sequencing.

### Subcellular localization of BNYVV p31 in *N. benthamiana* leaves using confocal laser scanning microscopy (CLSM)

Binary vectors were transformed into the *Agrobacterium tumefaciens* strain EHA105, and agrobacterium infiltrations of *N. benthamiana* leaves were performed essentially as described by Johansen and Carrington (2001). The agrobacterium mixtures also usually included bacteria containing the pGD-P19 plasmid to minimize host gene silencing [72]. The lower epidermal cells of agro-infiltrated leaves were assayed by CLSM at 3 dpi. Fluorescence analysis was performed using a Nikon ECLIPSE TE2000-E inverted fluorescence microscope equipped with a Nikon D-ECLIPSE C1 spectral confocal laser scanning system. RFP was excited using a 543 nm laser and imaged using the META detector set for 570–600 nm. DAPI fluorescence was excited with a filter set consisting of an emission filter of 435–485 nm and a 408-nm laser. The images were captured and processed with ECLIPSE EZ-C1 3.00 FreeViewer software, and data were captured as single optical sections.

### cDNA library preparation and Illumina deep sequencing

Total mRNA was purified using oligo (dT) magnetic beads and fragmented according to the instructions provided by the manufacturer (Illumina, San Diego, CA USA). The short fragments were then used as templates to synthesize first-stand cDNA using reverse transcriptase with random hexamer priming. After second-strand cDNA synthesis using DNA polymerase I, dNTPs, and RNase H, the products were amplified by PCR and purified after end-repair and removal of the ligation adaptors. Three paired-end cDNA libraries were constructed, with insert sizes ranging from 150 to 250 bp. The cDNA libraries were sequenced on the Illumina sequencing platform (HiSeq 2000) using a 100-bp paired-end approach, and the raw reads generated by Soleaxa/Illumina sequencing were submitted to the Sequence Read Archive database at NCBI, Accession No. SRA065289.

### Reads assembly and transcriptome annotation

*N. benthamiana* draft genome information was downloaded from the sol genomics network (http://solgenomics.net/organism/Nicotiana_benthamiana/genome). After removal of all adaptor sequences, empty reads, and low quality reads (Q<30, length <50 bp), the processed clean reads were mapped to the reference genome using the spliced aligner Tophat v2.0.6 [73] by allowing up to two mismatches and reporting up to 40 alignments. The resulting alignments were assembled into unigenes using Cufflinks software v2.0.2 [73]. Finally, the unigene sequences were aligned by BLASTX (an E-value<1.0e^–5^ was used as the cut-off) to public protein databases that included the NCBI non-redundant database (NR), Swiss-Prot database, Cluster of Orthologous Groups (COG) database, and Kyoto Encyclopedia of Genes and Genomes (KEGG) database. The proteins with the highest sequence similarity were retrieved for analysis. COG matched each annotated sequence to an ancient conserved domain to represent major phylogenetic lineages and KEGG enabled annotations of metabolic pathways. Based on NR annotation, Gene Ontology annotations and further functional classification of unigenes were determined with the Blast2go (http://www.blast2go.org/) and InterProScan V5 (http://www.ebi.ac.uk/Tools/pfa/iprscan/) tools. KEGG pathway analyses were performed using the online KEGG Automatic Server (KAAS) (http://www.genome.jp/tools/kaas/).

### Analysis of differentially expressed genes

The number of mapped clean reads for each unigene was normalized to provide an FPKM value to evaluate the expressed value and quantify transcript levels. The Cuffdiff program of the Cufflinks software suite was used to identify genes that were significantly differentially expressed under different conditions. False discovery rate (FDR) was applied to identify the threshold of the P value in multiple tests and analyses of the genes. Differentially expressed genes were subjected to GO enrichment analyses using agriGO (http://bioinfo.cau.edu.cn/agriGO/) as described previously [74]. For metabolic pathway enrichment analysis, all differentially expressed unigenes were mapped to terms in the KEGG database and searched for KEGG terms that were significantly enriched after comparison with the whole transcriptome background.

### Quantitative Real-time PCR (qRT-PCR) validation

To confirm the results of transcriptome sequencing analysis, the relative mRNA expression levels of several randomly selected genes in RNA from mock-, BN3-, and BN34-infected leaves were evaluated. Three RNA samples were detected for each group; whereas one was the same as that used for RNA-seq analysis, the other two replicates were derived from different plant samples. qRT-PCR was performed in 96-well plates using the CFX96 real-time PCR detection system (Bio-Rad, Hercules, CA, USA) with the following temperature program: 95°C for 15 s, followed by 40 cycles of 95°C for 15 s, and then annealing at 60°C for 30 s. For relative quantification of mRNA, 1 µg at total RNA was extracted from the leaves, treated with DNaseI (Takara) and reverse transcribed following the manufacturer's instructions. Each reaction mixture consisted of 1 µl cDNA, 7 µl SsoFast EyaGreen Supermix (Bio-Rad, Hercules, CA, USA), 1.5 µl (3 pmol/µl) of both forward and reverse primers, and 3 µl PCR-grade water, as recommended by the manufacturer (TaKaRa, Dalian, China). Each reaction included amplification of *PP2A* transcripts, which provided an internal reference. All primers used in this study are listed in Table S2. All PCR experiments were performed in triplicate and the results were calculated using the CFX Mange Version 1.6 software (Bio-Rad, Hercules, CA, USA) with the default parameters.

## Supporting Information

Figure S1 **Histogram showing gene ontology (GO) classification.** The functions of unigenes are divided into three main categories: biological processes, cellular components, and molecular functions. In total, 19,045 unigenes with BLASTX matches were assigned to gene ontology.
(TIF)

Figure S2 **Histogram classifying clusters of orthologous groups (COG).** A total of 7,967 unigenes were grouped into 25 COG categories.
(TIF)

Table S1 **Length distribution of assembled transcripts.**
(DOCX)

Table S2 **Summary of functional annotations of non-redundant unigenes.**
(DOCX)

Table S3 **Unigenes KEGG pathway analysis.** (XLC).
(XLSX)

Table S4 **Primers used in qRT-PCR and construction of clones.** (XLC).
(XLSX)

File S1 **Annotation of public databases searches.** This table shows the results of searches of reference sequences using BLASTX against the NCBI NR, GO and KEGG databases with a cut-off E-value of 10^−5^. (XLC).
(XLSX)

File S2 **Differentially expressed transcripts among the three libraries.** (XLC).
(XLSX)
