# Supplementary figures and images for: Deep Sequencing–Based Transcriptome Profiling Reveals Comprehensive Insights into the Responses of Nicotiana benthamiana to Beet necrotic yellow vein virus Infections Containing or Lacking RNA4
Source: PLoS One. 2014 Jan 9;9(1):e85284. doi: 10.1371/journal.pone.0085284 (PMC3887015; doi:10.1371/journal.pone.0085284)

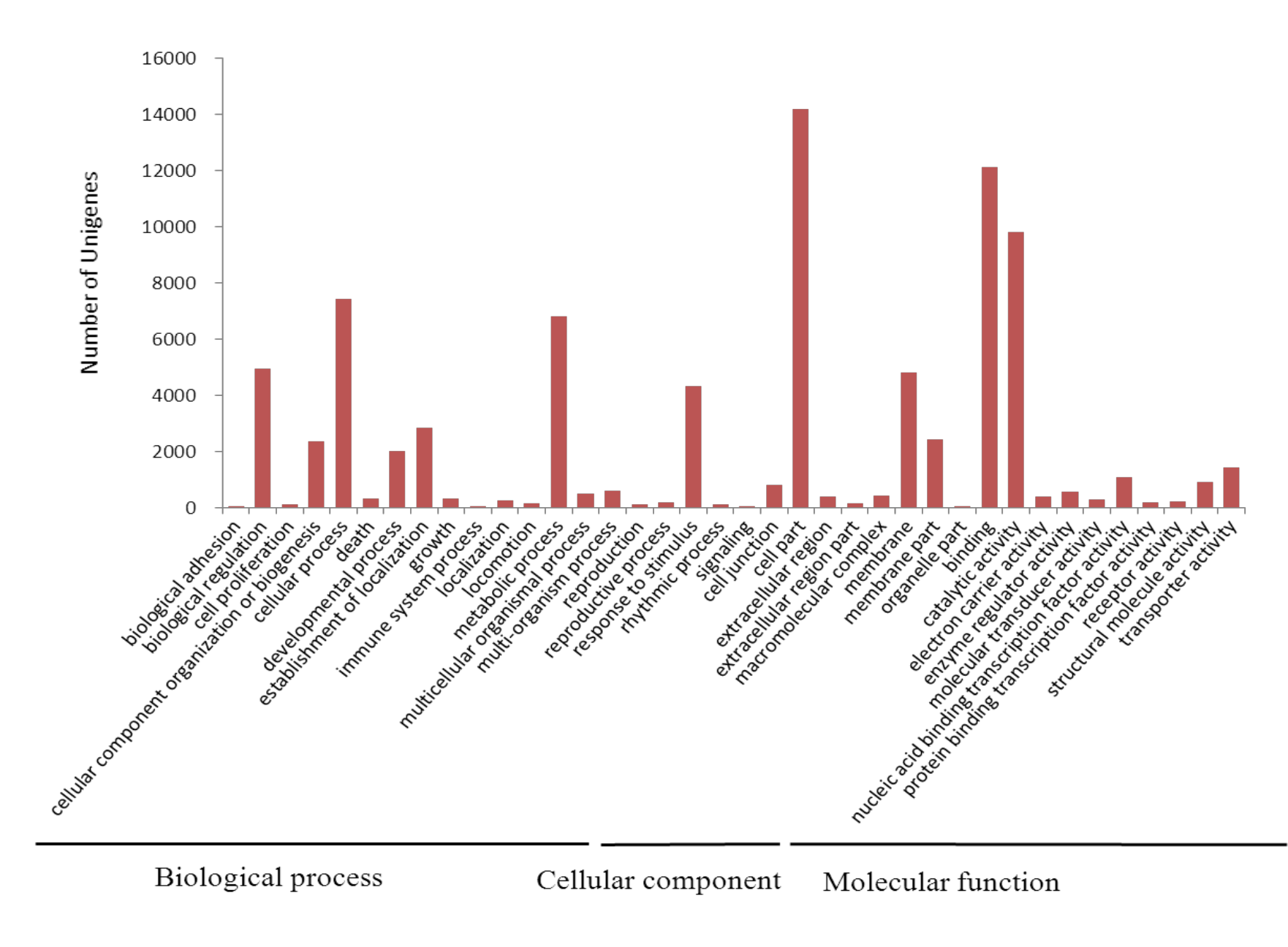

Supplement: Figure S1 — Histogram showing gene ontology (GO) classification. The functions of unigenes are divided into three main categories: biological processes, cellular components, and molecular functions. In total, 19,045 unigenes with BLASTX matches were assigned to gene ontology. (TIF) [file pone.0085284.s001.tif]

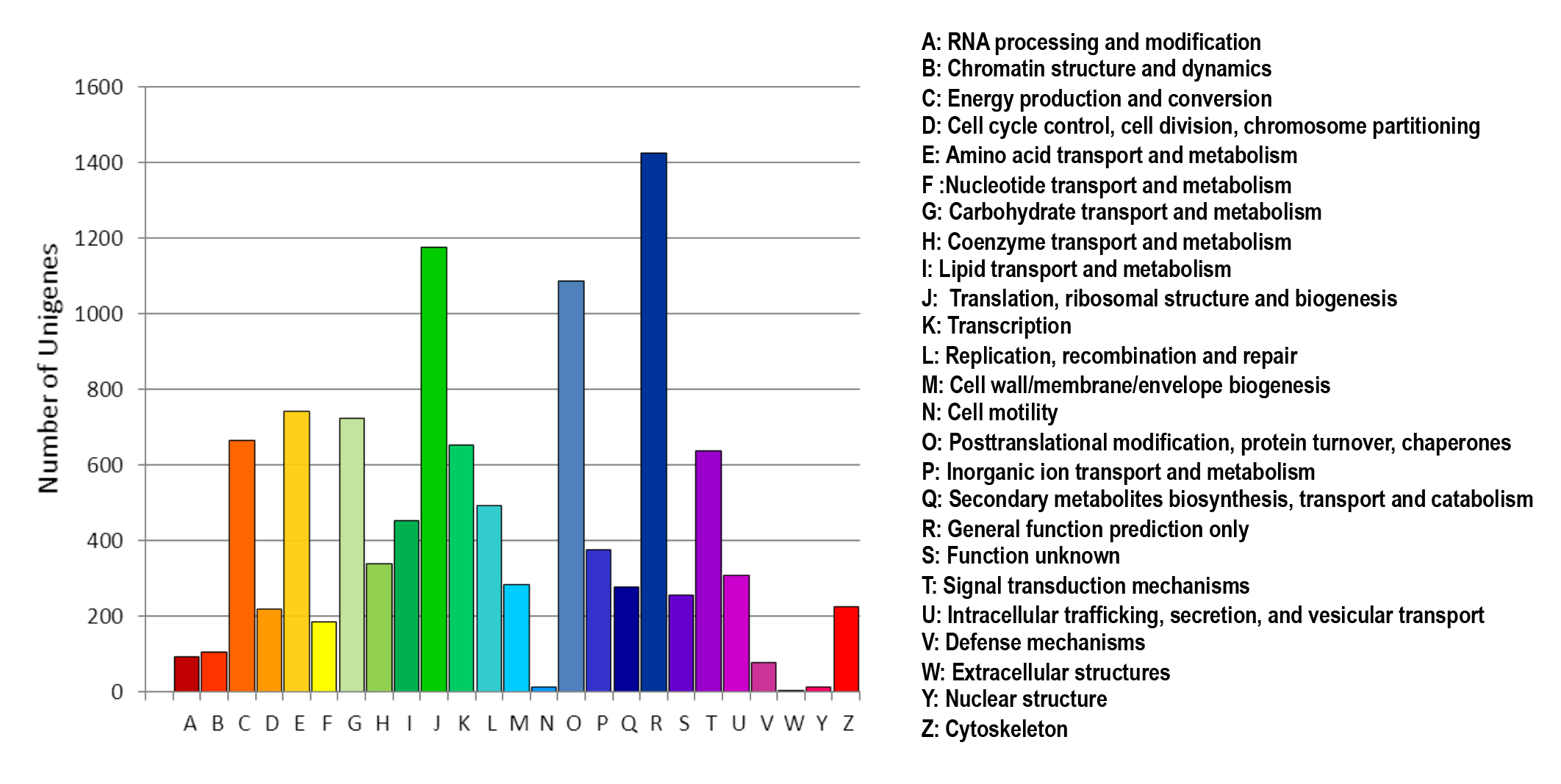

Supplement: Figure S2 — Histogram classifying clusters of orthologous groups (COG). A total of 7,967 unigenes were grouped into 25 COG categories. (TIF) [file pone.0085284.s002.tif]
