# Supplementary material for: Deep Sequencing–Based Transcriptome Profiling Reveals Comprehensive Insights into the Responses of Nicotiana benthamiana to Beet necrotic yellow vein virus Infections Containing or Lacking RNA4
Source: PLoS One. 2014 Jan 9;9(1):e85284. doi: 10.1371/journal.pone.0085284 (PMC3887015; doi:10.1371/journal.pone.0085284)
Supplement: Table S1 — Length distribution of assembled transcripts. (DOCX) [file pone.0085284.s003.docx]

**Table S1** Length distribution of assembled transcripts

| Transcript length(bp) | Total number | Percentage (%) |
| --- | --- | --- |
| 100-200 | 499 | 1.66% |
| 200-300 | 2,428 | 8.08% |
| 300-400 | 3,417 | 11.37% |
| 400-500 | 3,815 | 12.69% |
| 500-600 | 3,508 | 11.67% |
| 600-700 | 2,999 | 9.98% |
| 700-800 | 2,406 | 8.01% |
| 800-900 | 1,884 | 6.27% |
| 900-1000 | 1,570 | 5.22% |
| 1000-2000 | 6,470 | 21.53% |
| ≥2000 | 1,059 | 3.52% |
| Total transcripts number | 30,055 | 100% |
| Total length (bp) | 23,744,963 |  |
| N50 | 966 |  |
| Mean length (bp) | 790 |  |
| GC | 43.03(%) |  |
