# Supplementary material for: Deep Sequencing–Based Transcriptome Profiling Reveals Comprehensive Insights into the Responses of Nicotiana benthamiana to Beet necrotic yellow vein virus Infections Containing or Lacking RNA4
Source: PLoS One. 2014 Jan 9;9(1):e85284. doi: 10.1371/journal.pone.0085284 (PMC3887015; doi:10.1371/journal.pone.0085284)
Supplement: Table S2 — Summary of functional annotations of non-redundant unigenes. (DOCX) [file pone.0085284.s004.docx]

**Table S2** Summary of functional annotations of non-redundant unigenes

| **Database** | **27,890 all-unigenes(total annotated 24,024)** | |
| --- | --- | --- |
|  | **Number of annotated unigenes** | **Percentage of annotated unigenes** |
| **NR** | 24,005 | 86.07% |
| **KEGG** | 9,272 | 33.24% |
| **GO** | 19,045 | 68.15% |
| **COG** | 7,967 | 28.56% |
